# Prevalence and associated factors of strabismus in former preterm and full-term infants between 4 and 10 Years of age

**DOI:** 10.1186/s12886-017-0605-1

**Published:** 2017-12-02

**Authors:** Achim Fieß, Ruth Kölb-Keerl, Alexander K. Schuster, Markus Knuf, Bernd Kirchhof, Philipp S. Muether, Jacqueline Bauer

**Affiliations:** 1 Department of Ophthalmology, Helios Dr. Horst Schmidt Klinik, Ludwig-Erhard-Straße 100, 65199 Wiesbaden, Germany; 2 Department of Ophthalmology, University Medical Center Mainz, Mainz, Germany; 3 Department of Paediatrics, Helios Dr. Horst Schmidt Klinik, Wiesbaden, Germany; 4 Department of Ophthalmology, University of Cologne, Cologne, Germany

**Keywords:** Esotropia, Exotropia, Low birth weight, Low gestational age, Premature birth, Prevalence of strabismus

## Abstract

**Background:**

Limited data exist collating most of the associated factors for strabismus in one analysis. The aim of this study was to assess the prevalence of strabismus and to analyse associated factors in former preterm and full-term infants.

**Methods:**

In this cross-sectional study, 239 former preterm infants with gestational age (GA) ≤ 32 weeks and 264 former full-term born infants with GA ≥ 37 weeks underwent detailed ophthalmologic examination in the age of 4–10 years and perinatal data assessment for risk factor analysis. Ophthalmologic examinations included cover testing, best corrected visual acuity, cycloplegic objective refraction, slit lamp as well as fundus examinations. For association analysis with strabismus, the following data was collected and included in multivariable analysis: sex, age at examination, anisometropia, myopic and hyperopic refractive error (≥ 3 dioptres), astigmatism, birth weight percentile, gestational age, retinopathy of prematurity occurrence, maternal age at childbirth, mother smoking, breastfeeding < 3 months, artificial ventilation, intraventricular bleeding, and other perinatal adverse events.

**Results:**

Overall, 4/264 (2%) full-term infants, 15/125 (12%) preterm-infants with GA 29–32 weeks without ROP, 13/59 (22%) preterm infants with GA ≤ 28 weeks without ROP and 14/55 (26%) with GA ≤ 32 weeks with retinopathy of prematurity were affected by strabismus. In the multivariable regression model strabismus was associated with GA (OR = 0.84 per week; *p* = 0.001), hyperopic refractive error (OR = 4.22; *p* = 0.002) and astigmatism (OR = 1.68; *p* = 0.02).

**Conclusion:**

This investigation highlights that low gestational age and refraction of the eye are independent risk factors for strabismus, while the other factors show less independent influence.

## Background

In very preterm infants with low gestational age (GA) and low birth weight the prevalence of strabismus has been reported to be up to 42% [1–8]. Furthermore, its clinical effects in this population have important long-term consequences as well as public health significance. Infants developing strabismus are at increased risk for reduced visual acuity and for not developing binocular function during infancy and early childhood [9–13]. Therefore, infants with risk for strabismus have to be detected to prevent future visual dysfunction.

As main risk factors for strabismus low GA and reduced birth weight have been repeatedly discussed in the literature [5, 9, 12, 14–18]. Furthermore, in several studies other risk factors for strabismus were detected such as anisometropia [8, 9, 12, 19, 20], refractive error [7–9], astigmatism [9], cerebral palsy, intraventricular bleeding [12], parental age [16], maternal cigarette smoking during pregnancy [16, 21], low postnatal Apgar scores [22], presence of retinopathy of prematurity (ROP) [8, 23], and assisted delivery [24]. Scarce data exist collating most of the risk factors for strabismus in one analysis and to compare its effect on strabismus development in infants aged 4 to 10 years. Furthermore, a lack of data exist analysing risk factors for the different types of strabismus like esotropia and exotropia, separately. Due to an increasing survival rate of extremely preterm infants in recent years the significance of this topic is growing [25, 26], and has to be further explored.

The objective of this investigation was to assess the prevalence of strabismus in a large cohort of former preterm infants compared to full-term infants aged between 4 and 10 years. Additionally, the aim was to analyse most of the reported risk factors in one model to explore and identify independent risk factors. We will report on strabismus in general and stratify our analysis on esotropia and exotropia.

## Methods

### Subjects

The prospective controlled Wiesbaden Prematurity Study (WPS) was performed in former preterm and full-term infants born during the years of 2004 to 2010 in the Helios Dr. Horst Schmidt Klinik (tertiary centre of maximum care with level IV neonatology) in Wiesbaden, Germany between July 2014 and March 2015. The research was performed in accordance with the Declaration of Helsinki, and was approved by the Ethic Committee of the Physician Chamber Hessen. Written informed consent was obtained from all parents or legal guardians prior to examination.

### Recruitment methods

Inclusion criteria were (1) GA ≤ 32 weeks or ≥ 37 weeks, and (2) actual age between 4 and 10 years. The exclusion criteria were the presence of severe congenital anomalies since birth. Severe congenital anomalies were defined as infants with congenital chromosomal disorders or/and congenital heart diseases, or/and neural tube defects. Preterm and full-term infants were identified by our hospital database. Parents of all preterm infants and parents of randomly selected full-term infants received a phone call invitation to participate in our investigation.

### Assessment of pre- and postnatal history

For the enrolled children, history data were assessed from each child’s recorded file. Occurrences of perinatal and postnatal complications, such as intraventricular bleeding were assessed. Furthermore, occurrence of periventricular leukomalacia, necrotizing enterocolitis and sepsis were documented and summarized as perinatal adverse events. In addition, artificial ventilation and oxygen demand were analysed. Gestational age and birth weight were recorded and percentile of birth weight was calculated. According to German guidelines, postnatal ROP screening was initiated at 6 weeks after birth with regular follow-up until full retinal vascularization or until ROP activity regression after expected date of birth was achieved. Diode laser photocoagulation was performed if treatment for ROP was necessary [27].

For the period after hospital discharge all parents received the standard recommendation of close ophthalmic follow-up at a local eye care provider (ophthalmologist) in accordance to the current guidelines for premature infants [27]. No routine control appointments were fixed in our ophthalmological department except if the patients were sent from their local ophthalmologist.

### Ophthalmologic examination

All examinations were performed using a standardized protocol for paediatric eye examinations. Testing of best-corrected visual acuity was performed with Lea symbols until school enrolment and after that, with Landolt rings in all subjects. In cases of visual acuity below 20/200, depending on infant’s age Lea symbols or Landolt rings were used at a distance of 1 m. Values were converted for the analysis into the logarithm of the minimum angle of resolution (logMAR) [28].

Cyclopentolate (0.5%) eye drops were administered 3 times at 10-min intervals, after which cycloplegic refraction and keratometry were analysed with Nikon Nidek ARK-1 s keratometer (NIDEK CO., LTD., Gamagori, Japan). The spherical equivalent (refractive error) was calculated by adding the spherical value and half of the cylindrical value. Anisometropia was defined as a difference between the patients’ eyes of ≥ 1.5 dioptres of spherical equivalent.

### Orthoptic examination

Orthoptic examination for strabismus included the cover-uncover test and alternate cover test, Hirschberg-Test and examination of fixation behaviour, as well as presence or absence of nystagmus after having corrected refractive errors. If a child presented with heterotropia, an alternating prism cover test was added to measure the squint angle in dioptres. Strabismus was defined as constant or intermittent heterotropia of any dimension at distance and/or near fixation after having corrected refractive error. Classification of strabismus was categorized depending on deviation from primary position (esotropia, exotropia). Binocular function was tested by Lang-test. In addition, all parents were interviewed using a standardized protocol to request information concerning medical history of the child and parents, including ocular and general morbidities, as well as parental-specific information (mother age at childbirth, history of smoking, etc.). Additionally, a detailed ophthalmologic examination including measurements of slit lamp examination and funduscopy, assessment of ocular movements, as well as digital fundus photography was implemented. For history of ROP and ophthalmic measurements data of the right eye was included [29].

### Statistical analysis

Continuous variables were expressed as the mean ± standard deviation (SD) or as the median when appropriate. Categorical variables were expressed as proportions. The chi-square test was used to analyse the association between categorical variables. Normal distribution was tested with the Kolmogorov-Smirnov test. Mann-Whitney U test was used to compare independent continuous parameters between two groups and the Kruskal-Wallis test between several different groups. Two statistical models were used for risk factor analysis. First, separate univariate logistic regression analysis was performed with presence of strabismus (esotropia/exotropia) as dependent variable and documented potential risk factors for strabismus as independent variables, namely sex, gestational age (weeks), birth weight percentile, age at examination, hyperopic and myopic refractive error (categorial; for spherical equivalent ≥ +3D and for ≤ -3D)), anisometropia (categorical ≥ 1.5D), astigmatism (continuous), presence of ROP, maternal age at childbirth, mother smoking before birth, breastfeeding < 3 months, artificial ventilation, intraventricular bleeding, and other perinatal adverse events (periventricular leukomalacia, necrotizing enterocolitis, and sepsis). Second, we performed a multivariable logistic regression analysis to identify independently associated factors including all of the above-described factors that were associated with a *p*-value < 0.20 in the univariate analysis. The 95% confidence interval and odds-ratio are given. A sensitivity analysis was performed taking into account maximum ROP stage of both eyes. Collinearity was assessed calculating pairwise Spearman correlation coefficients. This is an explorative analysis, a *p*-value < 0.05 was considered as statistically significant. Calculations were performed using IBM SPSS 20.0 (SPSS Inc., Chicago, USA).

## Results

### Patient characteristics

**Fig. 1:**
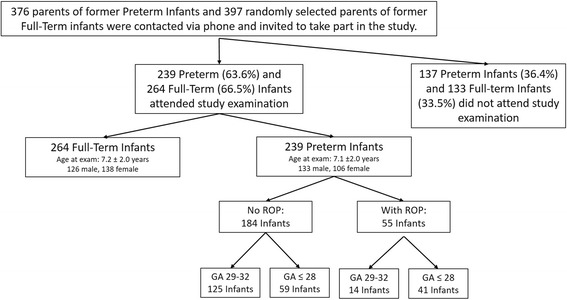
This figure presents the number of contacted parents and the number of participating infants for each group

Overall, 376 parents of former preterm infants and 397 randomly from the local registry of births selected parents of former full-term infants were contacted via phone and invited to take part in this study. Of these, 239 children born prematurely and 264 children born at full-term accepted to attend the clinical examination. In accordance, response rate was 63.6% for former preterm and 66.5% for former full-term born infants (Fig. 1). Of those, 264 were former full-term infants with a GA ≥ 37 weeks (group 1), 125 had a GA between 29 to 32 weeks without ROP (group 2), 59 had a GA ≤ 28 weeks without ROP (group 3), and 55 had a GA ≤ 32 weeks with postnatal ROP occurrence (group 4). Seven infants of the ROP group underwent laser treatment. Overall, 259 (51.5%) children were male. Of the 55 infants in group four with ROP, 14 infants had a GA between 29 and 32 weeks with 12 of them having a history of ROP stage 1 and two infants of ROP stage 2. Analyses of age, gender, birth characteristics, peri- and postnatal outcomes, medical record data, as well as information from parental interviews, are displayed for all groups in Table 1. Only 3/239 (1.3%) of all preterm infants reported that they have not had an ophthalmic examination after hospital discharge at a local eye care provider (ophthalmologist).

**Table 1:** Patient demographics, pre- and postnatal histories and parental interview information for full term neonates with GA ≥ 37 weeks (group 1), preterm infants of GA between 29 and 32 weeks without ROP (group 2), preterm infants of GA ≤ 28 weeks without ROP (group 3), and preterm infants with ROP occurrence after birth and GA ≤ 32 weeks (group 4)

	Group 1 ≥ 37 wks	Group 2 29–32 wks no ROP	Group 3 ≤ 28 wks no ROP	Group 4 ≤ 32 wks ROP
	*n* = 264	*n* = 125	*n* = 59	*n* = 55
Gestational age (weeks) ± SD	38.8 ± 1.4	30.5 ± 1.1	26.5 ± 1.3	26.7 ± 2.2
Gestational age (weeks) range	37–43	29–32	24–28	23–30
Birth weight (g) ± SD	3252 ± 545	1519 ± 382	963 ± 223	909 ± 331
Age at examination (years) ± SD	7.2 ± 2.0	7.1 ± 1.8	6.6 ± 2.2	7.6 ± 2.2
Male (*n* (%))	126 (47.7%)	72 (57.6%)	31 (52.5%)	30 (54.5%)
Multiparity (*n* (%))	20 (7.6%)	51 (40.8%)	15 (25.4%)	18 (32.7%)
ROP Stage (0/1/2/3/4)	(264/0/0/0/0)	(125/0/0/0/0)	(59/0/0/0/0)	(0/31/13/10/1)
Laser treatment for ROP (*n* (%))	0 (0%)	0 (0%)	0 (0%)	7 (12.7%)
Mean maternal age at childbirth (years) ± SD	30.8 ± 4.8	32.8 ± 5.5	31.7 ± 5.9	31.5 ± 6.0
Mother smoking (*n* (%))	38 (14.4%)	18 (14.4%)	10 (16.9%)	14 (25.5%)
Breastfeeding < 3 month (*n* (%))	86 (32.6%)	61 (48.8%)	35 (59.3%)	34 (61.8%)
Artificial ventilation (*n* (%))	5 (1.9%)	32 (25.6%)	46 (78%)	41 (74.5%)
Artificial ventilation (days) ± SD	2.0 ± 0.7	3.2 ± 2.5	13.2 ± 16.1	23.1 ± 22.6

Variables are expressed as means ± standard deviation (SD). Parts of this table are published elsewhere [37]. *wks* weeks of gestational age, *ROP* retinopathy of prematurity, *n* number of infants, *g* gram

The distribution and parameters of visual acuity, spherical equivalent, astigmatism, and anisometropia for the different groups are displayed in Table 2. It was noticeable that a higher proportion of infants in the ROP group showed myopic values ≥3 dioptre compared to the other three groups (*p* < 0.001).

**Table 2:** Distribution and parameters for each group for visual acuity, spherical equivalent, myopia, hypermetropia, astigmatism, anisometropia, strabismus, esotropia, exotropia, and nystagmus

	Group 1 ≥ 37 wks	Group 2 29–32 wks no ROP	Group 3 ≤ 28 wks no ROP	Group 4 ≤ 32 wks ROP	*p*-value
	*n* = 264	*n* = 125	*n* = 59	*n* = 55	
Visual acuity (logMAR) ± SD	0.01 ± 0.03	0.02 ± 0.10	0.05 ± 0.12	0.14 ± 0.33	< 0.001
Spherical equivalent (D) ± SD	1.1 ± 1.3	1.3 ± 1.5	1.2 ± 2.5	0.7 ± 3.3	0.046
SE range (D) (min/max)	(−5.25/+9.5)	(−9.75/+5.75)	(−12.0/+10.25)	(−11.5/+8.75)	
Myopia of −3 D or more (eyes (%))	2 (0.8%)	1 (0.8%)	1 (1.7%)	7 (13.0%)	<0.001
Hypermetropia (≥ 3 D) (eyes (%))	22 (8.3%)	12 (9.6%)	7 (12.1%)	5 (9.3%)	0.84
Mean cylinder per dioptre ± SD	0.4 ± 0.5	0.5 ± 0.6	0.7 ± 0.6	0.8 ± 1.0	< 0.001
Astigmatism (≥ 1.5 D) (eyes (%))	12 (4.5%)	7 (5.6%)	8 (13.8%)	8 (14.8%)	0.008
Anisometropia (≥ 1.5 D) (*n* (%))	8 (3.0%)	5 (4.0%)	3 (5.2%)	5 (9.4%)	0.08
Strabismus (*n* (%))	4 (1.5%)	15 (12%)	13 (22%)	14 (25.5%)	< 0.001
Esotropia (*n* (%))	3 (1.1%)	9 (7.2%)	11 (18.6%)	11 (20%)	< 0.001
Exotropia (*n* (%))	1 (0.4%)	6 (4.8%)	2 (3.4%)	3 (5.5%)	0.020
Nystagmus (*n* (%))	0 (0%)	1 (0.8%)	1 (1.7%)	3 (5.5%)	0.004

Data of the right eye was included into analysis, except for strabismus and anisometropia. In 3 eyes assessment of best-corrected visual acuity and objective refraction was not possible. Parts of this table are published elsewhere [37]

*wks* weeks of gestational age, *ROP* Retinopathy of prematurity, *n* number of infants, *eyes* number of eyes, *SD* standard deviation, *SE* spherical equivalent, *D* Dioptre

Ophthalmic parameters such as strabismus and type of strabismus differed between the four groups (table 2). In groups one to four, 4/264 (1.5%), 15/125 (12%), 13/59 (22%), and 14/55 (25.5%) of children were affected by strabismus, respectively (*p* < 0.001). Strabismus was detected in 26/419 (6.2%) patients with a birth weight exceeding 1000 g, in 13/55 (24%) patients with a birth weight of 750 g to 1000 g and in 7/29 (24%) patients with a birth weight below 750 g (*p* < 0.001). Infants with esotropia had infantile strabismus, which was also true for infants with exotropia. All infants with manifest strabismus had no binocular function.

Regarding stages of ROP, strabismus was observed in 7/31 (23%) children with stage 1 ROP, in 2/13 (15%) with stage 2 ROP, in 4/10 (40%) with stage 3 ROP, and in 1/1 (100%) children with stage 4 ROP (*p* < 0.001). Strabismus was found in 4/7 (57%) subjects with postnatal laser treatment due to ROP.

Pathologic nystagmus was detected in one child (1%) with GA between 29 to 32 weeks without ROP, in one child (2%) with GA ≤ 28 weeks without ROP and three children (6%) with GA ≤ 32 weeks with postnatal ROP occurrence (*p* = 0.004) (table 2). Different functional ophthalmic disorders were observed in these 5 children, such as non-physiologic deviation and saccades. Three had postnatal cerebral bleeding and one had retinal detachment including the macula. Visual acuity was reduced with 0.5 logMAR in all these children and all of them had deterioration of binocular depth and strabismus.

### Analysis on strabismus

#### Strabismus

In the univariate analysis, an association was found between strabismus and low GA, low birth weight, higher refractive error (for spherical equivalent ≥+3D and for ≤-3D), higher astigmatism, high anisometropia (≥ 1.5D), postnatal ROP occurrence, breastfeeding for less than 3 months, artificial ventilation and intraventricular bleeding (Table 3). No association with strabismus was present for percentile of birth weight, sex, age at examination, maternal age at childbirth, maternal smoking and other perinatal adverse events (Table 3).

**Table 3:** Values of risk factors for subjects with and without strabismus, and odds ratio and confidence interval (95%) for development of strabismus

Strabismus	Description^a^		Univariate Analysis^b^		Multivariable analysis^c^	
	Characteristics in children with strabismus *n* = 46	Characteristics in children without strabismus *n* = 457	OR (95% CI)	*p*-value	OR (95% CI)	*p*-value
Gestational age [weeks]	28.7 ± 3.9	34.5 ± 5.3	0.80 [0.74; 0.86]	**< 0.001**	0.84 [0.76; 0.93]	**0.001**
Birth weight percentile	41.3 ± 27.6	41.0 ± 26.5	1.00 [0.99; 1.01]	0.93		
Sex [male]	26 (56.5%)	233 (51.0%)	0.80 [0.43; 1.47]	0.48		
Age at examination [years]	7.2 ± 2.1	7.1 ± 2.0	1.03 [0.88; 1.19]	0.72		
Anisometropia ≥1.5 D [yes]	7 (15.6%)	14 (3.1%)	5.80 [2.21; 15.24]	**< 0.001**	2.89 [0.87; 9.55]	0.08
Refractive error ≤ −3 [diopter]^d^	4 (8.7%)	7 (1.5%)	6.10 [1.71; 21.67]	**0.005**	1.43 [0.26; 7.94]	0.68
Refractive error ≥ +3 [diopter]^d^	12 (26.1%)	34 (7.5%)	4.37 [2.07; 9.21]	**< 0.001**	4.22 [1.70; 10.50]	**0.002**
Astigmatism [diopter]	0.93 ± 0.94	0.47 ± 0.54	2.16 [1.52; 3.09]	**< 0.001**	1.68 [1.07; 2.64]	**0.024**
ROP [yes]	14 (30.4%)	41 (9%)	4.44 [2.19; 8.99]	**< 0.001**	0.88 [0.35; 2.21]	0.78
Maternal age at childbirth [years]	31.1 ± 5.9	31.5 ± 5.3	0.99 [0.89; 1.10]	0.94		
Mother smoking [yes]	10 (21.7%)	70 (15.3%)	1.54 [0.73; 3.24]	0.26		
Breastfeeding < 3 months [yes]	28 (60.9%)	188 (41.1%)	2.23 [1.20; 4.14]	**0.012**	1.07 [0.52; 2.20]	0.85
Artificial ventilation [yes]	27 (58.7%)	97 (21.2%)	5.27 [2.81; 9.89]	**< 0.001**	1.40 [0.57; 3.44]	0.46
Intraventricular bleeding [yes,]	11 (23.9%)	26 (5.7%).	5.21 [2.38; 11.42]	**< 0.001**	2.08 [0.84; 5.18]	0.12
Other perinatal adverse events [yes]^e^	3 (6.5%)	11 (2.4%)	2.83 [0.76; 10.53]	0.12	0.95 [0.20; 4.42]	0.94

*n* number of infants, *OR* odds ratio, *CI* confidence interval, *ROP* retinopathy of prematurity; Data of the right eye was included in univariate and multivariable regression analysis for refractive error and astigmatism

^a^Variables are expressed as mean ± standard deviation (SD) or as number of children (n (%)), *P*-values below 0.05 were marked in bold

^b^based on univariate logistic regression analysis

^c^based on multivariable logistic regression model. Presented factors were included in the multivariable analysis when *P* value was < 0.20 in univariate analysis

^d^Reference was refractive error between −3 diopter and +3 diopter

^e^As perinatal adverse events were periventricular leukomalacia, necrotizing enterocolitis and sepsis summarized

Including factors with a *p* < 0.20 value in the univariate analysis, the multivariable logistic regression analysis showed independently associated factors with strabismus, namely low gestational age (OR = 0.84 per week; *p* = 0.001), hyperopic refractive error (for spherical equivalent ≥+3D) (OR = 4.22; *p* = 0.002), and astigmatism (OR = 1.68 per dioptre; *p* = 0.02) (Table 3).

#### Esotropia

In the univariate analysis, esotropia was associated low GA, low birth weight, higher refractive error (for spherical equivalent ≥+3D and for ≤-3D), high anisometropia (≥1.5D), higher astigmatism, postnatal ROP occurrence, artificial ventilation, intraventricular bleeding (Table 4). No association with strabismus was present for percentile of birth weight (percentile), sex, age at examination, maternal age at childbirth, maternal smoking, breastfeeding for less than 3 months, and other perinatal adverse events (Table 4).

**Table 4:** Values of risk factors for subjects with and without esotropia, and odds ratio and confidence interval (95%) for development of strabismus

Esotropia	Description^a^		Univariate Analysis^b^		Multivariable analysis^c^	
	Characteristics in children with strabismus *n* = 34	Characteristics in children without strabismus *n* = 469	OR (95% CI)	*p*-value	OR (95% CI)	*p*-value
Gestational age [weeks]	28.5 ± 4.0	34.4 ± 5.3	0.79 [0.73; 0.87]	**< 0.001**	0.85 [0.75; 0.96]	**0.009**
Birth weight percentile	45.2 ± 29.7	40.7 ± 26.3	1.01 [0.99; 1.02]	0.34		
Sex [male]	19 (55.9%)	240 (51.2%)	0.83 [0.41; 1.67]	0.60		
Age at examination [years]	7.2 ± 2.3	7.1 ± 2.0	1.02 [0.85; 1.21]	0.85		
Anisometropia ≥1.5 D [yes]	6 (18.2%)	15 (3.2%)	6.70 [2.41;18.63]	**< 0.001**	2.95 [0.84; 10.43]	0.09
Refractive error ≤ −3 [diopter]^d^	3 (8.8%)	8 (1.7%)	5.55 [1.40; 21.98]	**0.015**	1.52 [0.24; 9.48]	0.65
Refractive error ≥ +3 [diopter]^d^	12 (35.3%)	34 (7.3%)	6.95 [3.17; 15.23]	**< 0.001**	7.83 [2.88; 21.27]	**< 0.001**
Astigmatism [diopter]	0.93 ± 0.82	0.48 ± 0.57	2.04 [1.40; 2.96]	**< 0.001**	1.62 [0.98; 2.69]	0.06
ROP [yes]	11 (32.4%)	44 (9.4%)	4.62 [2.11; 10.10]	**< 0.001**	0.88 [0.30; 2.53]	0.81
Maternal age at childbirth [years]	30.4 ± 5.1	31.5 ± 5.3	0.96 [0.90; 1.03]	0.23		
Mother smoking [yes]	9 (26.5%)	71 (15.1%)	2.02 [0.90; 4.50]	0.09	1.87 [0.69; 5.05]	0.22
Breastfeeding < 3 months [yes]	20 (58.8%)	196 (41.8%)	1.99 [0.98; 4.04]	0.06	0.72 [0.29; 1.74]	0.46
Artificial ventilation [yes]	21 (61.8%)	103 (22%)	5.74 [2.78; 11.86]	**< 0.001**	2.01 [0.68; 5.91]	0.21
Intraventricular bleeding [yes]	9 (26.5%)	28 (6.0%).	5.67 [2.42;13.30]	**< 0.001**	2.47 [0.89; 6.79]	0.08
Other perinatal adverse events [yes]^e^	2 (5.9%)	12 (2.6%)	2.38 [0.51; 11.09]	0.27		

*n* number of infants, *OR* odds ratio, *CI* confidence interval, *ROP* retinopathy of prematurity; Data of the right eye was included in univariate and multivariable regression analysis for refractive error and astigmatism

^a^Variables are expressed as mean ± standard deviation (SD) or as number of children (n (%)), P-values below 0.05 were marked in bold

^b^based on univariate logistic regression analysis

^c^based on multivariable logistic regression model. Presented factors were included in the multivariable analysis when P value was < 0.20 in univariate analysis

^d^Reference was refractive error between −3 diopter and +3 diopter

^e^As perinatal adverse events were periventricular leukomalacia, necrotizing enterocolitis and sepsis summarized

In multivariable logistic regression analysis (Tab. 4), an independent association was observed for low gestational age (OR = 0.85 per week; *p* = 0.009) and higher refractive error (spherical equivalent ≥+3D) (OR = 7.83; *p* < 0.001) (Table 4).

#### Exotropia

In univariate analysis exotropia was associated with low gestational age, low birth weight and high astigmatism, while the other factors were not associated (Table 5). Multivariable logistic regression analysis was not conducted due to the small number of children with exotropia (Table 5).

**Table 5:** Values of risk factors for subjects with and without exotropia, and odds ratio and confidence interval (95%) for development of strabismus. Multivariable logistic regression analysis was not conducted due to the small number of children with exotropia

Exoptropia	Description^a^		Univariate Analysis^b^	
	Characteristics in children with strabismus *n* = 12	Characteristics in children without strabismus *n* = 491	OR (95% CI)	*p*-value
Gestational age [weeks]	29.3 ± 4.0	34.1 ± 5.4	0.84 [0.74; 0.95]	**0.006**
Birth weight percentile	30.3 ± 17.0	41.3 ± 26.7	0.98 [0.96; 1.01]	0.17
Sex [male]	7 (58.3%)	252 (51.3%)	0.75 [0.24; 2.41]	0.63
Age at examination [years]	7.3 ± 1.8	7.1 ± 2.0	1.06 [0.79; 1.41]	0.70
Anisometropia ≥1.5 D [yes]	1 (8.3%)	20 (4.1%)	2.13 [0.26; 17.29]	0.48
Refractive error ≤ −3 [diopter]^c^	1 (8.3%)	10 (2.0%)	4.36 [0.51; 37.04]	0.18
Refractive error ≥ +3 [diopter]^c^	0 (0%)	46 (9.4%)	0.00 [0.00; 0.00]	0.99
Astigmatism [diopter]	0.92 ± 1.3	0.5 ± 0.57	1.82 [1.07; 3.12]	**0.028**
ROP [yes]	3 (25.0%)	52 (10.6%)	2.81 [0.74; 10.72]	0.13
Maternal age at childbirth [years]	33.0 ± 7.5	31.4 ± 5.3	1.06 [0.95; 1.18]	0.31
Mother smoking [yes]	1 (8.3%)	79 (16.1%)	0.47 [0.06; 3.72]	0.48
Breastfeeding < 3 months [yes]	8 (66.7%)	208 (42.4%)	2.72 [0.81; 9.16]	0.11
Artificial ventilation [yes]	6 (50.0%)	118 (24.0%)	3.16 [1.00; 9.99]	0.050
Intraventricular bleeding [yes]	2 (16.7%)	35 (7.1%).	2.61 [0.55; 12.36]	0.23
Other perinatal adverse events [yes]^d^	1 (8.3%)	13 (2.6%)	3.34 [0.40; 27.85]	0.27

*n* number of infants, *OR* odds ratio, *CI* confidence interval, *ROP* retinopathy of prematurity; Data of the right eye was included in univariate and multivariable regression analysis for refractive error and astigmatism

^a^Variables are expressed as mean ± standard deviation (SD) or as number of children (n (%)), P-values below 0.05 were marked in bold

^b^based on univariate logistic regression analysis

^c^Reference was refractive error between −3 diopter and +3 diopter

^d^As perinatal adverse events were periventricular leukomalacia, necrotizing enterocolitis and sepsis summarized

#### Sensitivity analysis

The sensitivity analysis taking into account maximum ROP stage of both eyes revealed comparable results for strabismus and its associated factors.

## Discussion

Our results demonstrate that prevalence of strabismus is significantly increased in children born prematurely compared to children of the same age born at full-term. In the multivariable analysis an association of strabismus with low gestational age, higher refractive error (spherical equivalent ≥+3D) and astigmatism was observed, while prior reported risk factors could not be confirmed.

The highlight of this study is the assessment and the analysis of a broad range of prior reported risk factors for strabismus using a multivariable analysis to determine independently associated factors.

In numerous studies multiple risk factors for strabismus were detected [5, 7–9, 12, 14–24]. Nevertheless, previous studies explored different sub-sets of risk factors and do not always incorporate associations between these factors. The results of our study highlight the necessity of incorporation of this structure. In the univariate analysis several factors exposed a significant association with strabismus, while these factors were no longer associated in multivariable analysis in our large cohort. In multivariable analysis, only low GA, hyperopic refractive error and astigmatism were significantly associated with strabismus.

Few studies investigated the impact of low GA and low birth weight on strabismus development and reported different results [5, 9, 12, 14–18]. Two studies showed an increased risk for strabismus in preterm infants with low gestational age [5, 12], while two other large, retrospective, population-based cohort studies reported an association with low birth weight [14, 17]. Torp-Pederson et al. [14] reported also an association with prematurity while Gulati et al. [17] found no association between strabismus and GA when it was adjusted for birth weight. These studies concluded that a low birth weight (under 2000 g) is a more important factor for strabismus than GA. The limitations of these studies were the retrospective design and that various risk factors, such as refractive error, were not included in the multivariable analysis. In our analysis gestational age showed a strong association with strabismus, as did birth weight with strabismus. This indicates that both factors are valid and the more immature the infant is born the higher is the risk for strabismus. However, due to the strong correlation between gestational age and birth weight, we included birth weight percentile in our models which revealed no association in uni- and multivariable analysis indicating that low birth weight relative to gestational age is less important for strabismus.

Hyperopic refractive error (spherical equivalent ≥+3D) was found as an independent risk factor for strabismus and esotropia in our multivariable analysis. Similarly, in the Sydney Childhood Eye Study an association between increased hyperopia and strabismus was detected with a spherical equivalent of + 2.46 D in infants with strabismus compared to + 1.23 D in children without strabismus [9]. Moreover, the Multi-Ethnic Pediatric Eye Disease Study and the Baltimore Pediatric Eye Disease Study showed a linear correlation between refractive error and increasing presence of strabismus. Hypermetropia of 3 dioptre or more was found as the strongest predictor of esotropia [12]. This finding is in accordance with our results and underlines the importance of this parameter.

Robaei et al. [9] reported in a population based study of 6-year old infants that children with strabismus had a higher astigmatism (0.32 dioptres higher) compared to infants without strabismus. Our study shows comparable findings that astigmatism is independently associated with strabismus, independently of gestational age and hyperopic refractive error highlighting its aspect to strabismus development in childhood.

Overall, our results revealed a relatively low strabismus proportion for premature infants of up to 26% compared to other trials. The prevalence of strabismus in children varies between studies due to different inclusion criteria, different study designs and divergent characteristics of subjects. Strabismus proportions of up to 42% are reported in long-term studies in premature infants [1–8], while in the current literature the prevalence of strabismus in full-term infants is described to ranges from 0.7 to 9.9% [30, 31]. In our study, 1.5% of full-term infants and depending on gestational age, 12–26% preterm infants had strabismus, which are comparable proportions.

With respect to type of strabismus, namely esotropia and exotropia, a study by O’Connor et al. [8], reported similar proportions for esotropia and exotropia as we detected. Two other investigations confirmed that esotropia was the most frequent type of strabismus [2, 32]. Some authors conclude that injuries at the time of critical brain development are a decisive factor for development of the different types of strabismus [9, 33]. As brain injuries are more frequent in immature infants [34] they probably contribute to the higher amount of strabismus in those infants. The various types of strabismus pathogenesis have not yet been sufficiently investigated, as there is a lack of power in previous trials to find important factors for analysing risk factors of each type separately. Therefore, the results of the present study are of considerable importance indicating that slightly different factors cause different types of strabismus.

Pathologic nystagmus was noticed in 5/239 (2.1%) preterm infants. These results are in accordance with previous reports, demonstrating nystagmus prevalence ranging from 2% to 10% in first decade of life in premature infants. The absence of nystagmus in the full-term children is similarly to previous trials [4, 34]. The clinical consequences for children with nystagmus are severe and associated with reduced visual acuity as well as impaired stereopsis. Patients with nystagmus in the present study exhibited nystagmus of low amplitudes and high frequency. Brodsky and colleagues reported that this type of nystagmus is often detected in cases of white matter damage [35], which frequently occurs in premature infants [36]. This is probably a result of a higher rate of visual pathway injuries in immature infants [34].

The strengths of this investigation were the prospective controlled study design with a high number of participates and the large control group of full-term children. Another important factor was the availability of pre-, peri- and postnatal medical information from all children, which allowed a very detailed examination as well as an adjustment for different possible confounding factors. The strict standardization reduced the probability of examiner-dependent variances. Limitations of the study are the single-centre and hospital-based study design, which reduces the representativeness of our study. Our data is restricted to children who had survived preterm birth, although neonatal intensive care has increased this chance in the recent year. Although, the participation rate was relatively high (> 60%), there is a chance that particular those infants with poor health outcomes did not reattend to take part in our study and may therefore be underrepresented, a common phenomenon in studies with former preterm infants. Accordingly, our results cannot be transferred to the general population. A lack of masked observers may also affect our results. In addition, a restriction of our visual acuity assessment is the fact that converted logMAR values are not as valid as visual acuity values obtained by actually using a logMAR chart. This has to be considered when interpreting our data. Furthermore, as only seven children of the former preterm infants received laser treatment, this treatment was not analysed separately, but were grouped to all infants with ROP.

## Conclusions

In conclusion, our study highlights that the main determinants for strabismus are low gestational age, hyperopic refractive error, and astigmatimsus. Furthermore, we can provide as one of few studies data about risk factors for esotropia and exotropia: esotropia was independently associated with low gestational age and hyperopic refractive error, while exotropia was linked to gestational age and higher astigmatism.

## Abbreviations

D: Dioptre
GA: gestational age
OR: Odds Ratio
ROP: Retinopathy of prematurity
WPS: Wiesbaden Prematurity Study

## References

[1] VanderVeen DK, Coats DK, Dobson V, Fredrick D, Gordon RA, Hardy RJ, Neely DE, Palmer EA, Steidl SM, Tung B (2006). Prevalence and course of strabismus in the first year of life for infants with prethreshold retinopathy of prematurity: findings from the early treatment for retinopathy of prematurity study. Arch Ophthalmol.

[2] VanderVeen DK, Bremer DL, Fellows RR, Hardy RJ, Neely DE, Palmer EA, Rogers DL, Tung B, Good WV, Early Treatment for Retinopathy of Prematurity Cooperative G (2011). Prevalence and course of strabismus through age 6 years in participants of the early treatment for retinopathy of prematurity randomized trial. J AAPOS.

[3] Cats BP, Tan KE (1989). Prematures with and without regressed retinopathy of prematurity: comparison of long-term (6-10 years) ophthalmological morbidity. J Pediatr Ophthalmol Strabismus.

[4] Gallo JE, Lennerstrand G (1991). A population-based study of ocular abnormalities in premature children aged 5 to 10 years. Am J Ophthalmol.

[5] Schalij-Delfos NE, de Graaf ME, Treffers WF, Engel J, Cats BP (2000). Long term follow up of premature infants: detection of strabismus, amblyopia, and refractive errors. Br J Ophthalmol.

[6] Laws D, Shaw DE, Robinson J, Jones HS, Ng YK, Fielder AR (1992). Retinopathy of prematurity: a prospective study. Review at six months. Eye (Lond).

[7] Holmstrom G, Rydberg A, Larsson E (2006). Prevalence and development of strabismus in 10-year-old premature children: a population-based study. J Pediatr Ophthalmol Strabismus.

[8] O'Connor AR, Stephenson TJ, Johnson A, Tobin MJ, Ratib S, Fielder AR (2002). Strabismus in children of birth weight less than 1701 g. Arch Ophthalmol.

[9] Robaei D, Rose KA, Kifley A, Cosstick M, Ip JM, Mitchell P (2006). Factors associated with childhood strabismus: findings from a population-based study. Ophthalmology.

[10] Oliver M, Nawratzki I (1971). Screening of pre-school children for ocular anomalies. II. Amblyopia. Prevalence and therapeutic results at different ages. Br J Ophthalmol.

[11] Shaw DE, Fielder AR, Minshull C, Rosenthal AR (1988). Amblyopia--factors influencing age of presentation. Lancet.

[12] Cotter SA, Varma R, Tarczy-Hornoch K, McKean-Cowdin R, Lin J, Wen G, Wei J, Borchert M, Azen SP, Torres M (2011). Risk factors associated with childhood strabismus: the multi-ethnic pediatric eye disease and Baltimore pediatric eye disease studies. Ophthalmology.

[13] Repka MX, Summers CG, Palmer EA, Dobson V, Tung B, Davis B (1998). The incidence of ophthalmologic interventions in children with birth weights less than 1251 grams. Results through 5 1/2 years. Cryotherapy for retinopathy of prematurity cooperative group. Ophthalmology.

[14] Torp-Pedersen T, Boyd HA, Poulsen G, Haargaard B, Wohlfahrt J, Holmes JM, Melbye M (2010). Perinatal risk factors for strabismus. Int J Epidemiol.

[15] Williams C, Northstone K, Howard M, Harvey I, Harrad RA, Sparrow JM (2008). Prevalence and risk factors for common vision problems in children: data from the ALSPAC study. Br J Ophthalmol.

[16] Chew E, Remaley NA, Tamboli A, Zhao J, Podgor MJ, Klebanoff M (1994). Risk factors for esotropia and exotropia. Arch Ophthalmol.

[17] Gulati S, Andrews CA, Apkarian AO, Musch DC, Lee PP, Stein JD (2014). Effect of gestational age and birth weight on the risk of strabismus among premature infants. JAMA Pediatr.

[18] Cumberland PM, Pathai S, Rahi JS (2010). Prevalence of eye disease in early childhood and associated factors: findings from the millennium cohort study. Ophthalmology.

[19] Dobson V, Sebris SL (1989). Longitudinal study of acuity and stereopsis in infants with or at-risk for esotropia. Invest Ophthalmol Vis Sci.

[20] Huynh SC, Wang XY, Ip J, Robaei D, Kifley A, Rose KA, Mitchell P (2006). Prevalence and associations of anisometropia and aniso-astigmatism in a population based sample of 6 year old children. Br J Ophthalmol.

[21] Hakim RB, Tielsch JM (1992). Maternal cigarette smoking during pregnancy. A risk factor for childhood strabismus. Arch Ophthalmol.

[22] Mohney BG, Erie JC, Hodge DO, Jacobsen SJ (1998). Congenital esotropia in Olmsted County, Minnesota. Ophthalmology.

[23] Bremer DL, Palmer EA, Fellows RR, Baker JD, Hardy RJ, Tung B, Rogers GL (1998). Strabismus in premature infants in the first year of life. Cryotherapy for retinopathy of prematurity cooperative group. Arch Ophthalmol.

[24] Pathai S, Cumberland PM, Rahi JS (2010). Prevalence of and early-life influences on childhood strabismus: findings from the millennium cohort study. Arch Pediatr Adolesc Med.

[25] Spencer R (2006). Long-term visual outcomes in extremely low-birth-weight children (an American ophthalmological society thesis). Trans Am Ophthalmol Soc.

[26] Zin A (2001). The increasing problem of retinopathy of prematurity. Community Eye Health.

[27] Deutsche Ophthalmologische Gesellschaft, Berufsverband der Augenärzte Deutschlands, Gesellschaft für Neonatologie und Pädiatrische Intensivmedizin (2008). Guidelines for ophthalmological screening of premature infants. Ophthalmologe.

[28] Bach M, Kommerell G (1998). Determining visual acuity using European normal values: scientific principles and possibilities for automatic measurement. Klin Monatsbl Augenheilkd.

[29] Fan Q, Teo YY, Saw SM (2011). Application of advanced statistics in ophthalmology. Invest Ophthalmol Vis Sci.

[30] Goh PP, Abqariyah Y, Pokharel GP, Ellwein LB (2005). Refractive error and visual impairment in school-age children in Gombak District, Malaysia. Ophthalmology.

[31] Maul E, Barroso S, Munoz SR, Sperduto RD, Ellwein LB (2000). Refractive error study in children: results from La Florida, Chile. Am J Ophthalmol.

[32] Gursoy H, Basmak H, Bilgin B, Erol N, Colak E (2014). The effects of mild-to-severe retinopathy of prematurity on the development of refractive errors and strabismus. Strabismus.

[33] Dobbing J, Sands J (1973). Quantitative growth and development of human brain. Arch Dis Child.

[34] Lindqvist S, Vik T, Indredavik MS, Skranes J, Brubakk AM (2008). Eye movements and binocular function in low birthweight teenagers. Acta Ophthalmol.

[35] Brodsky MC, Fray KJ, Glasier CM (2002). Perinatal cortical and subcortical visual loss: mechanisms of injury and associated ophthalmologic signs. Ophthalmology.

[36] Skranes JS, Martinussen M, Smevik O, Myhr G, Indredavik M, Vik T, Brubakk AM (2005). Cerebral MRI findings in very-low-birth-weight and small-for-gestational-age children at 15 years of age. Pediatr Radiol.

[37] Fieß A, Kölb-Keerl R, Elflein H, Schuster AK, Knuf M, Kirchhof B, Oberacher-Velten I, Muether PS, Bauer J. Evaluation of ophthalmic follow-up care of former preterm and full-term infants aged from 4 to 10 years in Germany – results of the Wiesbaden prematurity study (WPS) Klin Monbl Augenheilkd. 2017. doi:10.1055/s-0043-118852. 10.1055/s-0043-118852 29117610

